# Absence of low back pain to demarcate an episode: a prospective multicentre study in primary care

**DOI:** 10.1186/s12998-016-0085-z

**Published:** 2016-02-18

**Authors:** Andreas Eklund, Irene Jensen, Malin Lohela-Karlsson, Charlotte Leboeuf-Yde, Iben Axén

**Affiliations:** Unit of Intervention and Implementation Research, Karolinska Institutet, Institute of Environmental Medicine, Nobels v 13, S-171 77 Stockholm, Sweden; Research Department, Spine Center of Southern Denmark, Institute for Regional Health Research, Hospital Lillebælt, University of Southern Denmark, Østre Hougvej 55, DK-5500 Middelfart, Denmark

**Keywords:** Low back pain, LBP, Definition, Recovery, Episode, Demarcation, Primary care, Chiropractic, Absence, Non-episode

## Abstract

**Background:**

It has been proposed that an episode of low back pain (LBP) be defined as: “a period of pain in the lower back lasting for more than 24 h preceded and followed by a period of at least 1 month without LBP”. Previous studies have tested the definition in the general population and in secondary care populations with distinctly different results. The objectives of this study (in a primary care population) were to investigate the prevalence of 1) the number of consecutive weeks free from bothersome LBP, 2) the prevalence of at least four consecutive weeks free from bothersome LBP at any time during the study period, and 3) the prevalence of at least four consecutive weeks free from bothersome LBP at any time during the study period among subgroups that reported >30 days or ≤30 days of LBP the preceding year.

**Method:**

In this prospective multicentre study subjects with LBP (*n* = 262) were consecutively recruited from chiropractic primary care clinics in Sweden. The number of days with bothersome LBP was collected through weekly automated text messages. The maximum number of weeks in a row without bothersome LBP and the number of periods of at least four consecutive weeks free from bothersome LBP was counted for each individual and analysed as proportions.

**Results:**

Data from 222 recruited subjects were analysed, of which 59 % reported at least one period of four consecutive weeks free from bothersome LBP. The number of consecutive pain free weeks ranged from 82 (at least one) to 31 % (9 or more). In subjects with a total duration of LBP of ≤ 30 days the previous year, 75 % reported a period of 4 consecutive weeks free from bothersome LBP during the study period whereas this was reported by only 48 % of subjects with a total duration of LBP of >30 days the previous year.

**Conclusion:**

Prevalence of four consecutive pain free weeks is found in the majority of subjects in this population logically reflects duration of LBP within the sample and may be applied on patients in primary care to demarcate a LBP episode.

## Background

Low back pain (LBP) is a prevalent condition [1, 2] often with an intermittent course [3, 4] with episodic flare-ups [5, 6] and periods without pain [7, 8]. A definition of what constitutes an episode of LBP is fundamental for the study of new episodes, risk factors, resolution, persistence and recurrence [9]. To specify when one episode ends and a new one begins, a period free from pain (in previous research described as a “non-episode” [7, 8]) is required. Recovery is a term that may be used to demarcate such a period with absence of pain following or preceding an episode of LBP. However, there is no evidence-based definition of recovery [10] to date. Such a definition would aid in the exploration of pain trajectories to subgroup individuals and possibly tailor interventions accordingly.

De Vet et al. [9] proposed a definition of an episode of LBP based on an extensive literature search and group discussions with researchers and clinicians. They proposed that an episode of LBP be defined as: “a period of pain in the lower back lasting for more than 24 h preceded and followed by a period of at least 1 month without LBP”. In a recent [11] modified Delphi approach, it was agreed to incorporate de Vet’s definition into the consensus definition of recovery.

Leboeuf-Yde et al. [7] investigated if part of de Vet’s proposed definition, namely “at least 1 month without LBP” was applicable in two populations of LBP patients from secondary care. Using weekly data, the prevalence of periods of at least four consecutive weeks free from bothersome LBP was determined. It was found that only 18 and 20 % of the patients reported at least one period of a minimum of four consecutive weeks free from bothersome LBP during the 1-year study period.

Leboeuf-Yde et al. proposed that a relationship could exist between duration of pain and the absence of pain. Thus, one would expect patients with LBP of shorter duration to have longer consecutive pain free periods compared to patients with LBP of longer duration. The above described method was thus repeated in a sample from the general population and the prevalence of at least 4 consecutive weeks free from bothersome LBP was, as expected, found to be much higher, 83 %, during the 1-year study period [8]. The authors concluded that it would be possible to use 1 month of absence of bothersome LBP as a measure in order to study the occurrence of episodes in the general population. Whether the definition is applicable in a primary care population has not been tested, and doing so could reveal if it can be used as a demarcation of an episode. A logical relationship between the prevalence of four consecutive pain free weeks and duration of LBP would be expected.

The use of text messages is a novel and promising data collection method [12] for clinical research, as most people have a mobile phone and carry it with them at all times. This enables repeated and frequent measures without recall bias. Text messages have been used in many different settings in the investigation of diseases [7, 8, 12] and behaviours [13, 14]. Further, this type of data allow for an in depth analysis of the fluctuation of pain.

This study utilizes weekly text message data [12, 15] and replicates the method from the previous studies [7, 8]. The aim is to investigate the applicability of de Vet et al.’s [9] definition (as a demarcation of an episode of LBP) in a primary care population by estimating prevalence of 4 consecutive weeks free from bothersome pain during the study period.

## Methods

### Design

The data for this prospective multicentre study with a 6 month follow up period were collected in Sweden between May 2008 and June 2009 [12] with the primary aim of describing the clinical course of LBP. This report is based on a secondary analysis of those data. To effectively recruit patients, chiropractic clinics were chosen as LBP is the most common condition treated by chiropractors in Sweden [16]. Thirty-five chiropractors recruited up to 10 consecutive LBP patients each. To ensure sufficient academic and clinical standards, only members from the Swedish Chiropractic Association (SCA) were invited. The involved clinicians were representative of SCA’s members in terms of age, sex, years in practice and have a geographical spread across Sweden with the highest densities around the major cities (Stockholm, Göteborg and Malmö) [12] .

### Objectives

The objectives were to study the six months prevalence of:

1. The number of consecutive weeks free from bothersome LBP.
2. At least 4 consecutive weeks free from bothersome LBP at any time during the study period.
3. At least 4 consecutive weeks free from bothersome LBP at any time during the study period among subgroups that reported having had altogether >30 days or ≤30 days of LBP the preceding year.

We hypothesised our population to reflect that subjects with a previous history of longer LBP duration (>30 days of LBP the preceding year) would report fewer consecutive pain-free weeks as compared to those with a previous history of shorter duration of their LBP (≤30 days of LBP the preceding year).

### Subjects and data

Subjects were recruited when they sought chiropractic care for non-specific LBP with or without leg pain. Participants were of working age and were excluded if pregnant, unable to understand Swedish, did not have a mobile phone, or were unable to send text messages from their mobile phones. Patients that had been under chiropractic care during the previous 3 months were also excluded as were those with specific LBP (i.e., where pathology was suspected or present). After receiving information about the study and signing informed consent forms, the study subjects filled in a baseline questionnaire with information on sex, age and occupation, as well as area, intensity, duration and frequency of the LBP. Treatment content was decided by the individual chiropractor and not regulated by the research protocol. To minimize the burden on the participating clinicians, data on screened but ineligible subjects were not collected. The data from this study is a secondary analysis of a convenience sample collected during 2008 (reported 2012). The recruitment and data collection process have been described in detail in a previous publication [12].

### Text messages

SMS-Track® is a web-based system designed specifically for research to enable frequent data collection using text messages [15]. Previous studies have shown this to be an inexpensive method [17] that yields high response rates [12, 18], and good compliance. Compliance is not affected by age, sex or season [12]. The system uses a web-based interface, which can be accessed in real time to monitor compliance.

### Measurements

The term “bothersomeness” has been used in previous studies as a measurement for the impact of pain [19–21]. The term has been shown to correlate well with self-rated health [22], pain intensity [18], disability, psychological health (anxiety, depression), prediction of future work absence and healthcare consultations [23], and has been suggested as a standard outcome measure in LBP research [21]. Choosing this term further aligns with the bio-psychosocial model of care [24, 25].

During the 6-month study period the subjects were monitored with a weekly text message asking: “How many days this previous week has your low back pain been bothersome?” (requiring an answer between 0 and 7, sent in a reply text message). The weekly data were used in this study to examine the definition of episode with regard to absence of bothersome LBP. To elicit information regarding previous duration, the study subjects were asked as part of the baseline questionnaire to state if they had experienced more or less than 30 days of pain during the past year. Previous research [26] has found this classification of duration to have prognostic value, as it predicts treatment outcome for chiropractic patients with LBP [26]. This classification has been used in number of studies [12, 18, 27–29] to subgroup LBP patients. Three possible answer categories were available in this study; ≤30 days; >30 days intermittently; >30 days with more or less daily pain. For the purpose of this study, the two latter choices were collapsed into one category.

### Ethical considerations

Participation was voluntary and all participants received information regarding the study and signed informed consent forms. Ethics approval was granted by the ethics committee at Karolinska Institutet, 2007/ 1458-31/4.

### Data analysis

The number of consecutive weeks free from LBP was counted using a programmed bespoke syntax in SPSS v20 (available from the authors upon request). To conform to one of the previous studies [7] only subjects who replied to the weekly text messages at least 50 % of the time were included to ensure reliable estimates. Missing cells were considered to be weeks with LBP in the main analysis (as in one of the previous studies [8]) in order to avoid overestimation of the presence of consecutive weeks free from LBP. To assess the possible bias due to the imputation method, a sensitivity analysis to illustrate best case scenario was performed where the missing data were considered to be weeks free from LBP.

To investigate the applicability of de Vet et al.’s definition of LBP episodes, three main data analyses were performed with regard to the absence of LBP over 6 months: 1) The maximum number of consecutive weeks free from LBP, 2) the prevalence of at least four consecutive weeks free from LBP at any time during the study period, and 3) the same analyses among the subpopulations that reported either >30 or ≤30 days of LBP the previous year.

The data are reported as percentages with 95 % confidence intervals.

## Results

A total of 262 subjects agreed to participate in the study but 18 (7 %) dropped out. Twenty-two subjects (8 %) replied to less than 50 % of the weekly text messages were excluded from the analyses. Overall the response rate was high and in the final dataset, 222 subjects were included (85 % of the source population providing 5772 data points) with a total of 654 (11 %) missing weekly data points. During week 1 and 26 there were 7 % (16 data points) and 15 % (33 data points) missing data respectively. Descriptive data of the study population are summarized in Table 1.

**Table 1:** Descriptive data of the study population

Variable	Dropouts	Excluded	Study sample
Number of subjects	18	22	222
Age in years, mean (SD^a^)	43 (11)	41 (14)	44 (11)
Sex distribution, %			
Men	56	64	50
Women	44	36	49
Main type of occupation, %:			
Sitting	38	29	43
Standing	13	19	22
Varying	37	28	28
Heavy	12	24	7
Pain intensity 0–10, mean (SD^a^)	3.9 (3.1)	5.4 (2.3)	4.3 (2.2)
Presence of leg pain, %	59	47	49
Pain duration >30 days, %	33	57	59

^a^ *SD* standard deviation

The distribution of the maximum number of consecutive LBP-free weeks in a row per individual is reported in Table 2. The vast majority, 82 % (CI: 71–93), experienced at least 1 week without LBP at some point during the study period. During the study, 59 % (CI: 51–67) of the subjects experienced at least one period of at least four consecutive weeks free from LBP.

**Table 2:** Distribution of weeks free from bothersome pain

Maximum number of consecutive weeks free from bothersome pain	% (95 % CI^a^) *N* = 222
0	18 (16–20)
1	9 (8–10)
2	5 (4–6)
3	9 (8–10)
4	8 (7–9)
5	6 (5–7)
6	5 (4–6)
7	4 (3–5)
8	5 (4–6)
9 or more	31 (27–35)

^a^ *CI* confidence interval

Among the 92 subjects with shorter duration LBP (≤30 days) the previous year, 75 % (CI: 60–90) reported a period of at least 4 consecutive weeks free from LBP during the study period. Within the group of 130 subjects who reported longer duration of LBP (>30 days) the previous year, 48 % (CI: 40–56) reported a period of at least 4 consecutive weeks free from LBP during the study period.

**Fig. 1:**
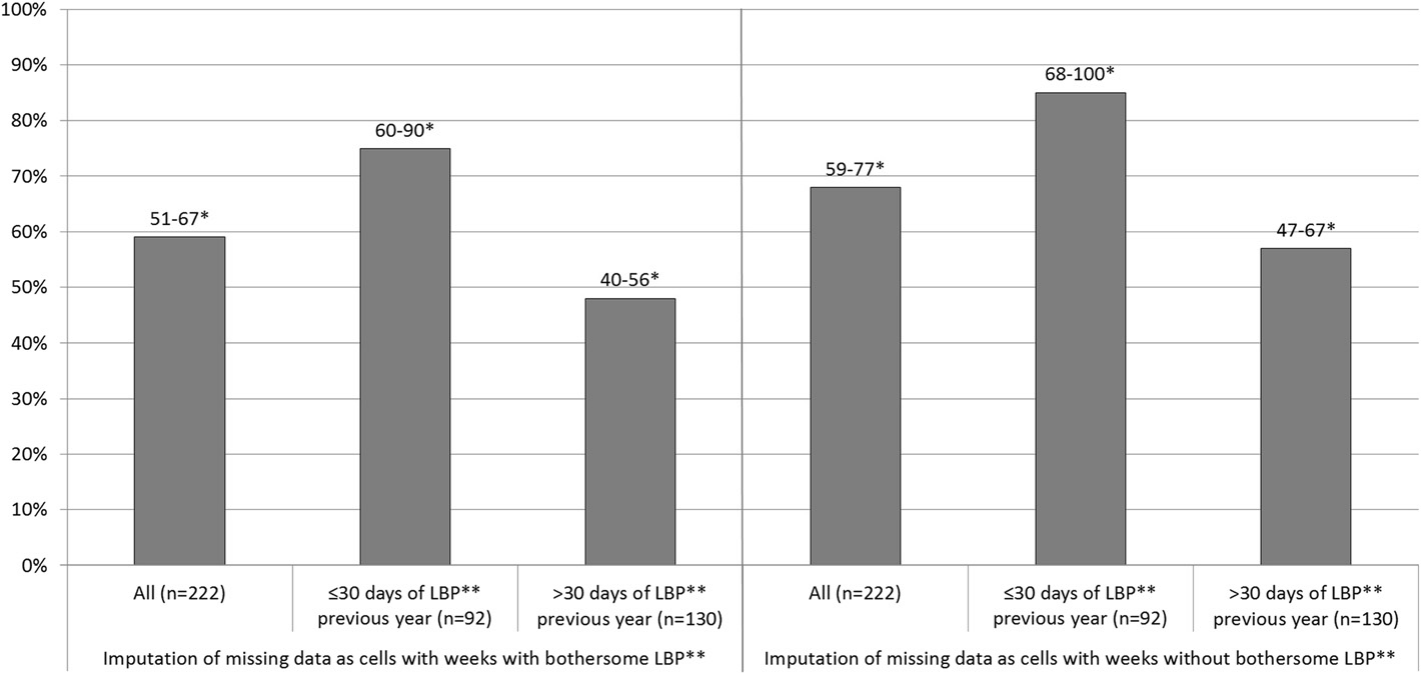
Sensitivity analysis comparing imputation of missing data as cells with or without weeks with bothersome pain in a best case and a worst case scenario. The Y-axis is displaying the percentage of the population with at least four consecutive weeks free from bothersome pain. *CI, Confidence Interval

The sensitivity analysis comparing the main analysis (worst case scenario) to a secondary analysis where missing data was imputed as weeks without LBP (best case scenario), showed a maximum difference of 10–15 % of consecutive weeks free from LBP across all measurements. See Fig. 1.

## Discussion

In line with de Vet et al’s definition, this is the first study to investigate the applicability of 4 weeks with absence of pain as a demarcation of an episode of LBP [9] in a primary care population. The results of this study support this definition as a demarcation of an episode in this population. Consecutive weeks free from pain occur at some point in a vast majority of our sample and inversely mirrors the duration of pain the previous year. The data support the use of de Vet’s definition as they reflect the expected variability of episodes in a primary care sample.

In this study, a third of the subjects reported 9 consecutive weeks free from LBP or more, while a fifth never reported a single week free from LBP. The presence of at least 4 consecutive weeks free from LBP at any time during the study period was found in a majority (82 %) of the subjects, i.e., it was more prevalent than in the secondary care populations [7], but less prevalent than the general population [8] (see Table 3). This relationship between a history of longer duration of pain and fewer consecutive weeks free from bothersome pain is confirmed when our sample is dichotomized according to previous duration. Thus prevalence of consecutive weeks free from pain seems to reflect previous duration of LBP in a logical manner.

**Table 3:** Comparison with other study samples from general population and secondary care

Variable	General population [8]	Chiropractic primary care population [This study]	Secondary care [7] (Two study samples)
Proportion of at least 4 consecutive weeks free from bothersome pain during the study period, % (95 % CI^a^)	83 (78–88)	59 (51–67)	20 (11–29), 18 (13–23)
Proportion women, %	54	49	68, 54
Age mean	50	44	46, 38
Pain intensity 0–10, mean	-	4.3	5.3, 4.9
Study period	12 months	6 months	12 months
Text message interval	Fortnightly	Weekly	Weekly

^a^ *CI* confidence interval

The high response rate and low recall bias of the repeated measures are the main strengths of the study and are a result of using weekly text messages [17]. Furthermore, the same data collection method and measurement were used in Leboeuf-Yde et al’s two previous studies [7, 8], which allows the results to be compared.

However, chiropractic subjects may differ somewhat from other primary care patients, perhaps limiting generalizability of the results. Recent research (2013) from Australia compared chiropractic patients to other patient groups in the primary care sector [30] and found that they are less disadvantaged but more likely to suffer from depression and other chronic health problems. This sample of patients has not received financial reimbursement of their chiropractic visits in contrast to other primary care consultations within the traditional Swedish healthcare system (where fees are normally subsidized). It is therefore possible that these patients differ in socioeconomic class compared to other primary care populations, which in turn may have resulted in a different psychological profile (possibly higher self-efficacy and better general health) compared to patients seen by physiotherapists or in general medical practice. Given the aim of the study, this does not pose a major problem as this particular population was selected specifically because it is likely to be different to those investigated in the previous two studies [7, 8].

The use of mobile-phones may pose a risk of selection bias across age groups. However the penetration of such technology is widespread in Sweden and during 2008 it was used by 94 % of the population [31]. Further, this study involved only people of working ages. Therefore this is not considered a source for bias in this study.

In Sweden, there are two different professional chiropractic organisations. All members of the SCA have been educated at an accredited institution (outside of Sweden) and hold an academic degree. The SCA members are probably not representative of all chiropractors in Sweden. However, the SCA members were chosen for reasons of comparability with other clinicians with similar academic standard within and outside of Sweden (such as Denmark where the two previous studies were conducted [7, 8]).

Missing data were treated as days with pain to avoid overestimating the LBP free periods. Similar to the study from the secondary care sector [7], only subjects who responded to more than 50 % of text messages were included which resulted in a limited number of missing values. Because of the high response rate, and good compliance, the sensitivity analysis showed only minor changes in the results and did not change the interpretation of the results.

The fact that “bothersomeness” was used as a measure of LBP may also affect the generalizability of our data when comparing to other studies where the presence of even minor LBP has been used [32]. One may argue that in reporting bothersome LBP, the result may overestimate weeks without LBP. De Vet et al. [9] explicitly refer to the “presence of pain” and reject “disabling pain” in the operational definition. However, although bothersomeness incorporates function it also closely correlates with pain intensity [18, 23]. Therefore, “bothersome pain” is distinctly different to “disabling pain” by also capturing pain that is not disabling but still relevant for the individual. Therefore, the deviation from de Vet et al’s operational definition is deemed reasonable, should not raise any methodological issues and should result in the reporting of mainly relevant levels of LBP. This study, along with the other two in this field [7, 8], is in fact using a more comprehensive term for LBP [9]. However, future research may investigate the correlation of consecutive weeks free from pain with other outcomes such as pain intensity, activity limitation, self-rated health and psychological factors.

Comparing the results from the chiropractic primary care patients to the previous studies from secondary care [7] and the general population [8] posed one potential major limitation. The data were collected during different follow-up periods, 6 months in our study compared to 12 months in the others, which limits the direct comparability between the cohorts and may have resulted in an underestimation of four consecutive weeks free from LBP in the present study population.

Research has shown that individuals with LBP may be clustered in specific trajectories [27]. Future research may investigate the usefulness of the duration of absence of pain as another variable that could be added to identify trajectories.

Four consecutive weeks free from LBP may also be useful as an outcome measure in clinical studies. A positive outcome may thus be defined in terms of frequency and duration of episodes of being free from LBP. Future research should test 4 consecutive weeks free from LBP for further clinical relevance and value as an outcome measure.

## Conclusions

A logical relationship exists between the prevalence of four consecutive pain free weeks and the study population, it being most common in the general population, followed by the primary care population and least common in the secondary care sector Further, absence of LBP is less common in patients from the primary care sector with a previous long duration of pain than in those with previous shorter pain duration. Therefore, a period of four consecutive pain free weeks may be applied both for research purposes and in clinical practice to demarcate a LBP episode.

## References

[1] Vassilaki M, Hurwitz EL (2014). Insights in public health: perspectives on pain in the low back and neck: global burden, epidemiology, and management. Hawaii J Med Public Health.

[2] Hoy D, Brooks P, Blyth F, Buchbinder R (2010). The epidemiology of low back pain. Best Pract Res Clin Rheumatol.

[3] Dunn KM, Jordan K, Croft PR (2006). Characterizing the course of low back pain: a latent class analysis. Am J Epidemiol.

[4] Chen C, Hogg-Johnson S, Smith P (2007). The recovery patterns of back pain among workers with compensated occupational back injuries. Occup Environ Med.

[5] Von Korff M (1994). Studying the natural history of back pain. Spine.

[6] Von Korff M, Saunders K (1996). The course of back pain in primary care. Spine.

[7] Leboeuf-Yde C, Jensen RK, Axen I (2012). Absence of low back pain in patients followed weekly over one year with automated text messages. Chiropr Man Therap.

[8] Leboeuf-Yde C, Lemeunier N, Wedderkopp N, Kjaer P (2014). Absence of low back pain in the general population followed fortnightly over one year with automated text messages. Chiropr Man Therap.

[9] de Vet HC, Heymans MW, Dunn KM, Pope DP, van der Beek AJ, Macfarlane GJ (2002). Episodes of low back pain: a proposal for uniform definitions to be used in research. Spine.

[10] Kamper SJ, Stanton TR, Williams CM, Maher CG, Hush JM (2011). How is recovery from low back pain measured? A systematic review of the literature. Eur Spine J.

[11] Stanton TR, Latimer J, Maher CG, Hancock MJ (2011). A modified Delphi approach to standardize low back pain recurrence terminology. Eur Spine J.

[12] Axen I, Bodin L, Bergstrom G, Halasz L, Lange F, Lovgren PW (2012). The use of weekly text messaging over 6 months was a feasible method for monitoring the clinical course of low back pain in patients seeking chiropractic care. J Clin Epidemiol.

[13] Smith KL, Kerr DA, Fenner AA, Straker LM (2014). Adolescents just do not know what they want: a qualitative study to describe obese adolescents’ experiences of text messaging to support behavior change maintenance post intervention. J Med Internet Res.

[14] Park LG, Howie-Esquivel J, Chung ML, Dracup K (2014). A text messaging intervention to promote medication adherence for patients with coronary heart disease: a randomized controlled trial. Patient Educ Couns.

[15] SMS-Track. https://www.sms-track.com/.

[16] Leboeuf-Yde C, Hennius B, Rudberg E, Leufvenmark P, Thunman M (1997). Chiropractic in Sweden: a short description of patients and treatment. J Manipulative Physiol Ther.

[17] Johansen B, Wedderkopp N (2010). Comparison between data obtained through real-time data capture by SMS and a retrospective telephone interview. Chiropractic & Osteopathy.

[18] Kongsted A, Leboeuf-Yde C (2010). The Nordic back pain subpopulation program: course patterns established through weekly follow-ups in patients treated for low back pain. Chiropr Osteopat.

[19] Cherkin DC, Deyo RA, Battie M, Street J, Barlow W (1998). A comparison of physical therapy, chiropractic manipulation, and provision of an educational booklet for the treatment of patients with low back pain. N Engl J Med.

[20] Daltroy LH, Cats-Baril WL, Katz JN, Fossel AH, Liang MH (1996). The North American spine society lumbar spine outcome assessment Instrument: reliability and validity tests. Spine.

[21] Deyo RA, Battie M, Beurskens AJ, Bombardier C, Croft P, Koes B (1998). Outcome measures for low back pain research. A proposal for standardized use. Spine.

[22] Patrick DL, Deyo RA, Atlas SJ, Singer DE, Chapin A, Keller RB (1995). Assessing health-related quality of life in patients with sciatica. Spine.

[23] Dunn KM, Croft PR (2005). Classification of low back pain in primary care: using “bothersomeness” to identify the most severe cases. Spine.

[24] Gatchel RJTD (1999). Psychosocial factors in pain: critical perspectives.

[25] Weiner BK (2008). Spine update: the biopsychosocial model and spine care. Spine.

[26] Leboeuf-Yde C, Axen I, Jones JJ, Rosenbaum A, Lovgren PW, Halasz L (2005). The Nordic back pain subpopulation program: the long-term outcome pattern in patients with low back pain treated by chiropractors in Sweden. J Manipulative Physiol Ther.

[27] Axen I, Bodin L, Bergstrom G, Halasz L, Lange F, Lovgren PW (2011). Clustering patients on the basis of their individual course of low back pain over a six month period. BMC Musculoskelet Disord.

[28] Axen I, Bodin L (2013). The Nordic maintenance care program: the clinical use of identified indications for preventive care. Chiropr Man Therap.

[29] Leboeuf-Yde C, Rosenbaum A, Axen I, Lovgren PW, Jorgensen K, Halasz L (2009). The Nordic Subpopulation Research Programme: prediction of treatment outcome in patients with low back pain treated by chiropractors-does the psychological profile matter?. Chiropr Osteopat.

[30] French SD, Densley K, Charity MJ, Gunn J (2013). Who uses Australian chiropractic services?. Chiropr Man Therap.

[31] Jönsson C (2008). The Swedish population’s use of the internet and telephones - an individual survey 2.

[32] McGorry RW, Webster BS, Snook SH, Hsiang SM (2000). The relation between pain intensity, disability, and the episodic nature of chronic and recurrent low back pain. Spine.

